# Temporal changes in blood–brain barrier permeability and cerebral perfusion in lacunar/subcortical ischemic stroke

**DOI:** 10.1186/s12883-015-0468-0

**Published:** 2015-10-22

**Authors:** Jun Yang, Christopher d’Esterre, Stefano Ceruti, Gloria Roversi, Andrea Saletti, Enrico Fainardi, Ting Yim Lee

**Affiliations:** Imaging Program, Lawson Health Research Institute, London, ON Canada; Imaging Research Lab, Robarts Research Institute, Western University, 1151 Richmond St. N, London, ON N6A 5B7 Canada; Radiology Department and Calgary Stroke Program, University of Calgary, Calgary, AB Canada; Neuroradiology Unit, Department of Neurosciences and Rehabilitation, Azienda Ospedaliero-Universitaria di Ferrara, Ferrara, Italy; Section of Neurology, Department of Medical and Surgical Sciences of the Communication and Behaviour, University of Ferrara, Ferrara, Italy

**Keywords:** Blood–brain barrier, Cerebral blood flow, Lacunar/subcortical infarct, CT perfusion, Cerebral small vessel disease

## Abstract

**Background:**

Cerebral microvascular abnormality is frequently associated with lacunar and subcortical ischemic lesions. We performed acute and follow-up CT perfusion scans over the first 3 months after ischemic stroke to investigate disturbances of the blood–brain barrier (BBB) and cerebral perfusion in patients with lacunar/subcortical lesions compared to those with cortical lesions alone.

**Methods:**

Thirty-one patients with lacunar/subcortical infarct (*n* = 14) or with cortical large vessel infarct (*n* = 17) were recruited and underwent a CT perfusion study at admission, 24 h, 7 days and 3 months after stroke using a two-phase imaging protocol. Functional maps of BBB permeability surface area product (BBB-PS), cerebral blood flow (CBF) and blood volume (CBV) at follow-up were co-registered with those at admission, and the measurements in non-infarcted ipsilateral basal ganglia and thalamus were compared within each group and between the two groups.

**Results:**

For the lacunar/subcortical group, BBB-PS within non-infarcted ipsilateral basal ganglia and thalamus peaked at day 7 compared to all other time points, and was significantly higher than the cortical group at day 7 and month 3. The CBF and CBV in the same region were significantly lower at admission and transient hyperemia was seen at day 7 in the lacunar/subcortical group.

**Conclusion:**

Disturbed BBB-PS and compromised cerebral perfusion over the first 3 months post stroke were shown in the non-infarcted basal ganglia and thalamus of lacunar/subcortical stroke using CT perfusion. Future studies are required to elucidate the relationship of post-stroke BBB disturbances to chronic cognitive impairment.

## Background

Stroke is one of the leading causes of death and long-term disability [1]. It is also an important contributing factor to cognitive dysfunction or dementia post stroke, including vascular cognitive impairment (VCI) [2]. Around 15–25 % of ischemic strokes are lacunar strokes [3], which can manifest as lacunes or subcortical lesions on routine MR and CT images. Recent clinical evidence has suggested that lacunar and subcortical lesion might exert adverse effects on cognition and memory [3–5]. Studies have shown that the blood–brain barrier (BBB) becomes more permeable in VCI patients with subcortical lesions and leukoaraiosis [6–8]. Contrast-enhanced MRI reveals that BBB permeability increases in patients with lacunar lesions, compared to normal control or cortical stroke [7–10]. Moreover, pathological studies report increased level of cerebrospinal fluid (CSF) albumin in patients with lacunar/subcortical lesion or white matter disease [3, 6]. Clinically, the primary types of brain lesion in cerebral small vessel-related VCI are lacunar and subcortical lesions, which are caused by ischemia due to arteriolar occlusion (e.g. lenticulostriate arteries, recurrent artery of Heubner and thalamoperforating arteries). The anatomic regions corresponding to these vascular territories are basal ganglia, thalamus and surrounding white matter (involved in motor movement, cognition, learning, visual memory and signal processing) [11, 12]. The ischemic event/occlusion may play as a trigger for the BBB abnormality in the presence of cerebral small vessel disease. Together, the evidence suggests that there is an underlying association between lacunar/subcortical ischemic stroke and cerebral microvascular abnormality, thus longitudinal investigation of subcortical BBB permeability may better demonstrate BBB leakage and microvascular dysfunction in stroke patients with small subcortical ischemic lesions before progressing to VCI. The chronic BBB leakage may act as a contributor and predictor for long-term cognitive impairment and associated pathology.

Recently, CT perfusion (CTP), a physiologic imaging modality requiring intravenous injection of iodinated contrast agent to image blood flow and associated hemodynamic parameters [13], is used for diagnosis of acute ischemic stroke and vasospasm. CTP not only measures tissue perfusion but also vascular permeability surface product (PS), an indicator of BBB integrity and permeability [13, 14]. Current CTP technique is more accessible and faster to perform in clinical practice than MRI and xenon-perfusion CT [15], and is ideal for studies at acute and subacute stages of stroke.

In this study we sought to examine the time course of BBB permeability changes measured with CTP in patients from the acute phase to 3 months post stroke to determine whether BBB permeability of the non-infarcted ipsilateral basal ganglia and thalamus is different in patients with and without lacunar/subcortical lesion.

## Methods

### Subjects

Patients with clinically diagnosed acute ischemic stroke were consecutively and prospectively recruited from February 2009 to July 2011 at one institution. All patients were admitted to the Department of Neuroscience of the University of Ferrara within 6 h of stroke symptom onset. Patients with impaired renal function, contraindications to iodinated contrast agent, intracerebral hemorrhage at admission, brain stem infarct, previous stroke with clear deficits, missing CTP imaging at admission or any follow-up time points (24 h, 7 days and 3 months), severe motion artifacts in CTP imaging, pregnancy and age < 18 years were excluded. For this study, thirty-one patients who underwent non-enhanced CT (NECT) and two-phase CTP acquisition (2.5 min) at admission and all follow-up exams were included. The study was approved by the Committee for Medical Ethics in Research of the University of Ferrara and informed consent was obtained from all patients enrolled in the study.

All patients were diagnosed by an experienced neurologist (G.R.) who evaluated the clinical stroke symptoms at admission based on the National Institutes of Health Stroke Scale (NIHSS) [16]. Clinical outcome was assessed using the modified Rankin scale (mRS) at 3-month post stroke [17] and mRS ≤ 2 and > 2 were defined as good and poor outcome, respectively. Patients having lacunar/subcortical lesion (≤20 mm in diameter) on month-3 NECT images were separated from those without subcortical lesion (i.e. large vessel infarcts primarily in the cortical gray matter). Vascular risk factors including hypertension, diabetes, previous silent infarct, ischemic heart disease and thrombolytic treatment were documented.

### CT perfusion acquisition protocol and functional maps

CTP studies were performed at admission, 24 h, 7 days and 3 months post stroke. Prior to CTP scan, a NECT scan was performed to locate hypodense ischemic lesion. Each CTP scan started with an intravenous injection of 50 mL of iodinated contrast agent (Iomeron 300 mg/ml, Bracco Imaging SpA, Milan, Italy) at the rate of 4 mL/s, followed by 45 mL of saline flush at the same infusion rate. A 20-gauge catheter and cephalic vein were used in peripheral venous access for contrast injection. Each CTP acquisition used a two-phase protocol: eight 5 mm-thick slices covering a 40 mm section of the brain were scanned continuously for 45 s with images reconstructed at 0.5 s intervals and then scanned once every 15 s for another 105 s for a total acquisition time of 2.5 min. The scan parameters for both phases were 25 cm FOV, 80 kV, 100 mA, and 1 s per gantry rotation. BBB permeability-surface (BBB-PS), cerebral blood flow (CBF) and cerebral blood volume (CBV) maps were generated with the delay insensitive CT Perfusion software based on the modified Johnson-Wilson model (GE Healthcare, Waukesha, WI) [18, 19].

### Image registration and analysis

**Fig. 1:**
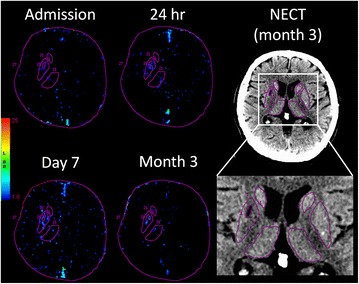
BBB-PS maps in a patient with a subcortical infarct in right putamen (as shown on the 3-month NECT). The maps from acute phase to 3 months after stroke were shown. Focally elevated BBB-PS was observed in right putamen at 24 h, day 7 and month 3 for the patient. Caudate nucleus, putamen, globus pallidus and thalamus in both ipsilateral and contralateral hemisphere were outlined in red. The infarct in the right putamen shown on the 3-month NECT was also outlined in red (smaller ROI within the right putamen)

CTP maps from all follow-up time points and NECT at 3 month of each patient were manually co-registered with the admission maps using Analyze v11.0 software (Mayo Clinic, Rochester, MN). The averages of the source CTP images were used as references for each registration. For lacunar/subcortical stroke, regions of interest (ROIs) were defined in the ipsilateral and contralateral basal ganglia (caudate nucleus, putamen, globus pallidus) and thalamus as well as the infarct using the month-3 NECT (Fig. 1). Data from the ipsilateral deep gray nuclei excluding the infarct were normalized with contralateral data to obtain relative CBF (rCBF), CBV (rCBV) and BBB-PS (rBBB-PS) for each time point. The same analysis was used for the cortical stroke group, except that no region of deep gray nuclei was excluded.

### Statistical methods

Statistical analyses were performed using SigmaPlot v12.0 (Systat Software, San Jose, CA). The unpaired t-test was used for comparisons of NIHSS score at admission, mRS at month 3 and age between patients with and without lacunar/subcortical lesion. Fisher’s exact test was used for demographic data between the two groups. Relative CBF, CBV and BBB-PS in the non-infarcted ipsilateral deep gray nuclei were compared between the two groups, and also between the time points within each group using two-way ANOVA with group and time as independent factors. Tukey’s post hoc test was then used for inter-group comparison. Statistical significance was set at *p* < 0.05. All CTP-derived data were presented as mean ± SEM.

## Results

The 31 patients (18 F, 13 M) included in this study were divided into two groups, 14 patients with lacunar/subcortical infarct and 17 patients with cortical stroke based on month-3 NECT images. Mean proportion of the infarcted area in the basal ganglia or thalamus for the lacunar/subcortical group was small, 11.4 ± 3.6 %. There were no significant differences in mean age, gender, hypertension and previous silent infarct between the two groups (Table 1). The proportion of the patients who received intravenous thrombolysis was lower but not significant in the lacunar/subcortical group (Table 1). The mean NIHSS at admission and mRS at 3 months post stroke were not significantly different between the two groups (Table 1).

**Table 1:** Characteristics of patients with and without lacunar/subcortical lesion

Clinical data	Subcortical/lacunar	Cortical	*p* value
	(*n* = 14)	(*n* = 17)	
Age^a^	71 ± 10	69 ± 12	0.50
Female n (%)	9 (64 %)	9 (53 %)	0.72
Hypertension n (%)	9 (64 %)	12 (71 %)	1.00
Previous silent infarct n (%)	5 (36 %)	7 (41 %)	1.00
Thrombolysis n (%)	10 (71 %)	14 (82 %)	0.67
NIHSS at admission^a^	15.1 ± 6.2	12.3 ± 6.2	0.21
mRs at month 3^a^	2.1 ± 1.1	2.2 ± 1.5	0.83

^a^Age, NIHSS and mRS are represented as mean ± SD

**Fig. 2:**
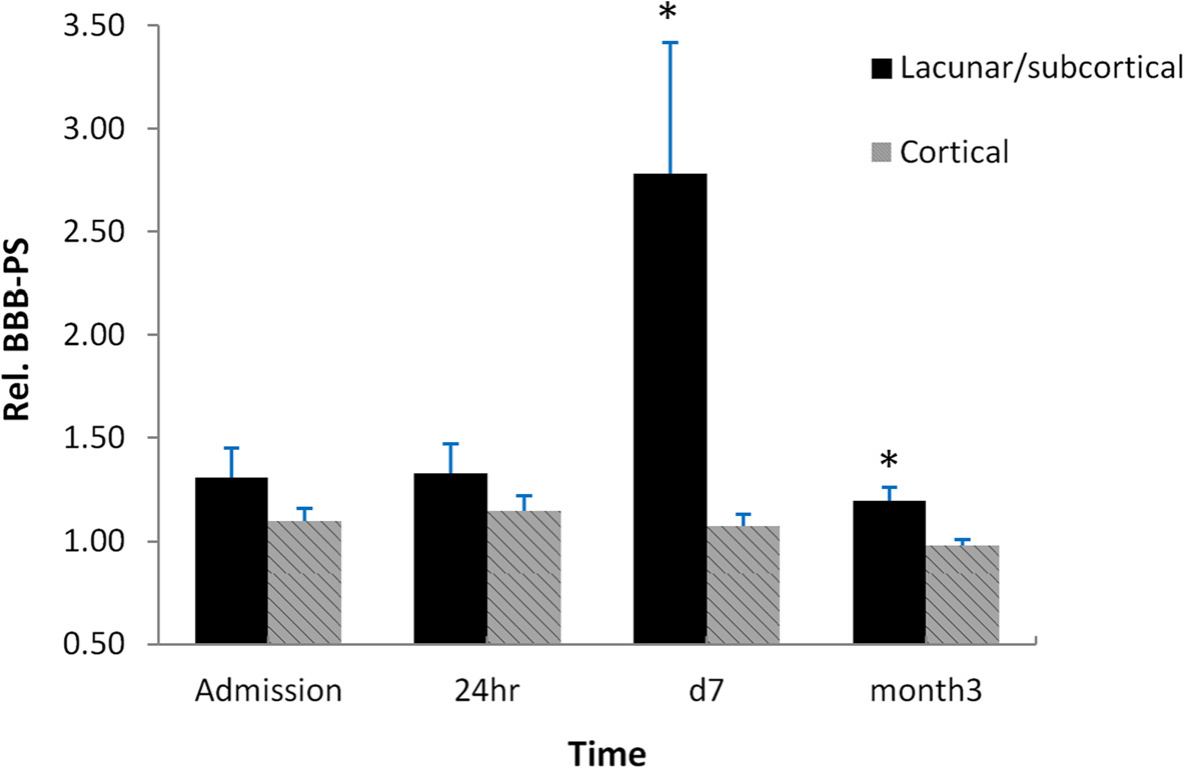
Blood–brain barrier (BBB) permeability (PS) in the non-infarcted ipsilateral basal ganglia and thalamus. rBBB-PS was significantly higher in the lacunar/subcortical group compared to the cortical group at 7 days and 3 months after stroke (*, *P* < 0.01 at 7 days and *P* < 0.05 at 3 months). The largest difference between the two groups occurred at day 7, with about 2.5-fold higher value in the lacunar/subcortical group than the cortical group. In the lacunar/subcortical group, rBBB-PS remained stable between admission and 24 h but significantly increased from 24 h to 7 days post stroke (*P* < 0.05), and then significantly declined at 3 months (*P* < 0.05). No significant intra-group differences in BBB-PS over time were seen in the cortical group

Mean rBBB-PS (Fig. 2) in the non-infarcted ipsilateral basal ganglia and thalamus (deep gray nuclei) in the lacunar/subcortical group was significantly higher at day 7 and month 3 (*p* < 0.01 at day 7 and *p* < 0.05 at month 3), and non-significantly higher at admission and 24 h than the cortical group. Particularly, at day 7 the lacunar/subcortical group showed the largest difference in rBBB-PS from the cortical group (2.78 ± 0.64 vs 1.07 ± 0.06). In addition, intra-group comparisons showed that the rBBB-PS within the lacunar/subcortical group at day 7 was significantly higher than those at all other time points (*p* < 0.05). This intra-group difference was not seen in the cortical patients. An example of increased BBB permeability was shown by the enhanced signal in BBB-PS maps (Fig. 1) in the right basal ganglia (putamen) where the contrast agent leaked into the interstitial space of brain tissue through a compromised BBB.

**Fig. 3:**
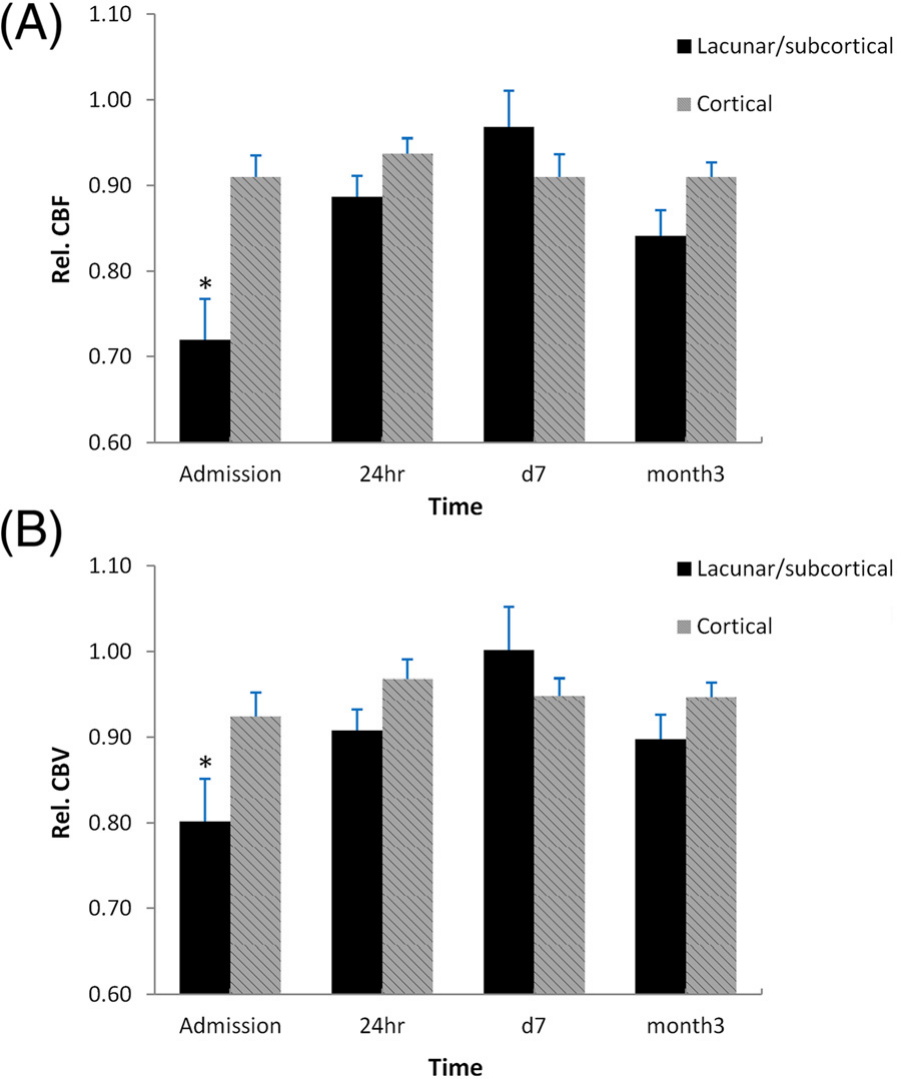
**a** CBF and **b** CBV in the non-infarcted ipsilateral basal ganglia and thalamus. Both rCBF and rCBV in the lacunar/subcortical group were significantly lower at admission (*, *P* < 0.01 for rCBF and *P* < 0.05 for rCBV) and remained lower at 24 h (no significance) than the cortical group. rCBF and rCBV were higher but not significant in the lacunar/subcortical group at day 7 compared to the cortical group. At month 3, there was no significant rCBF and rCBV difference between the two groups

Mean rCBF in the non-infarcted ipsilateral basal ganglia and thalamus (Fig. 3a) was significantly lower in patients with lacunar/subcortical lesions at admission, 0.72 ± 0.05, as compared to the cortical group, 0.86 ± 0.03 (*p* < 0.01). Similarly, mean rCBV in the non-infarcted ipsilateral basal ganglia and thalamus (Fig. 3b) in the lacunar/subcortical group was significantly lower than the cortical group at admission (0.80 ± 0.05 vs 0.92 ± 0.03, *p* < 0.05). There were no significant differences in both rCBF and rCBV at 24 h, day 7 and month 3 between the two groups, although at day 7 rCBF and rCBV were slightly higher in the lacunar/subcortical group.

## Discussion

In this study, CTP imaging revealed that patients with lacunar/subcortical lesions had significantly higher BBB-PS in the non-infarcted basal ganglia and thalamus at day 7 and month 3 than patients with cortical stroke. This finding is consistent with previous MRI evidence of increased BBB-PS in lacunar stroke and VCI with subcortical ischemic lesions [6–10, 20]. In addition to BBB-PS, at acute phase (admission) CBF and CBV within non-infarcted ipsilateral basal ganglia and thalamus in the lacunar/subcortical patients were significantly lower than the cortical group, suggesting an ischemic influence in the subcortical region.

Lacunar and subcortical lesions, along with white matter lesions (WML), are frequently found in patients with VCI and AD [6]. The well cited Nun study found that, in subjects with AD pathology, the presence of subcortical or lacunar infarcts (in the basal ganglia, thalamus and deep white matter) at autopsy was associated with a 20-fold higher risk to develop dementia compared to those without subcortical infarcts [21]. Therefore, it is important to understand the pathogenesis of lacunar/subcortical lesion. Some studies report that hypoperfusion (reduced CBF) was found in leukoaraiosis, which is frequently related to cerebral microvascular disturbances in lacunar/subcortical stroke [22, 23]. In our study, within the acute phase significantly reduced CBF and CBV was present in the non-infarcted basal ganglia and thalamus of the lacunar/subcortical patients, but not in the cortical patients, indicating the presence of a more severe ischemia in the basal ganglia and thalamus of the lacunar/subcortical group. At day 7, differences in CBF and CBV between the two groups were not significant but CBF and CBV were higher in the lacunar/subcortical group. This is probably due to reactive hyperemia or compensatory blood supply (reperfusion) from collateral flow to the viable penumbra, similar to the results found in animal model of cerebral ischemia [24]. This appeared to be transient since, at month 3, slightly lower CBF and CBV were again detected in the affected region in the lacunar/subcortical group possibly due to compromised vascular reactivity and tissue damage. Several studies show that greater BBB disruption is associated with reperfusion post cerebral ischemia in animal models [24–26]. This is consistent with our finding that the greatest BBB disruption (the highest BBB-PS) was observed along with the reperfusion at day 7 in lacunar/subcortical stroke. In addition, the majority of the patients in this study had thrombolytic treatment (tPA) at admission, which has also been associated with increased BBB permeability after reperfusion (as a secondary injury) in previous studies [27, 28]. Moreover, other reports indicate that early post-ischemia hyperperfusion may be associated with infarction or impaired BBB at later time [29, 30], which could explain the higher BBB-PS presented at month 3 in the lacunar/subcortical patients.

Hypoperfusion in the basal ganglia and thalamus at acute phase is not the only vascular abnormality of lacunar/subcortical lesion that is associated with cerebral small vessel disease. Additionally, increased BBB permeability (i.e. leaky cerebral microvessels) could be an underlying pathogenic mechanism that is exacerbated by ischemia [9, 10, 20]. Pathologically, extravasation of serum proteins such as albumin into CSF, which is an indicator of BBB disruption, has been demonstrated in VCI and AD patients, particularly in those with lacunar/subcortical ischemic lesion or WML [6, 7, 31]. In our study, BBB-PS in the basal ganglia and thalamus of the lacunar/subcortical patients was significantly elevated and peaked at day 7, compared to that of cortical group. This reflects a dynamic transition of BBB abnormality from acute phase to a maximum opening/disruption of BBB at subacute phase for lacunar/subcortical lesion. At month 3, BBB-PS of the non-infarcted basal ganglia and thalamus in the lacunar/subcortical group was still significantly higher than in the cortical group, but at a lower level than at day 7. This suggests that after an ischemic insult BBB in the viable area of the lacunar/subcortical group remained more affected and vulnerable than the cortical group. All these observations, to some extent, may explain early BBB-PS changes of cerebral microvascular disease, especially in the affected subcortical regions such as basal ganglia and thalamus, where about 31 and 12 % of lacunar infarcts are located respectively [11]. Recent perfusion MRI studies found significantly increased BBB permeability in the basal ganglia, CSF and deep white matter in subjects with VCI and lacunar/subcortical ischemic vessel disease [6–10, 20], which is consistent with our findings. This increased BBB permeability observed from our and other studies posits a link between cerebral small vessel disease and lacunar/subcortical lesions. All of the above evidence strongly supports cerebral microvascular dysfunction as an important contributing vascular mechanism for lacunar/subcortical lesions.

In comparison with previous studies [8–10], the strengths of this study include: (1) multiple time points post stroke at acute and subacute phase over the first 3 months to better identify early changes of BBB-PS, a biomarker of cerebral microvascular dysfunction. In addition, we registered CTP maps for all different time points to ensure measurements are from the same region; (2) multiple parameters such as CBF, CBV and BBB-PS can be produced at the same time for each CTP scan, increasing the chance to detect not only initial (acute) ischemic deficits with viable penumbra but also BBB disturbances at acute and subacute period; (3) in contrast to MRI, changes in CT signal intensity (attenuation) are linearly related to changes in contrast agent concentration, resulting in better measurements of perfusion parameters in detecting defects [14, 32].

The limitations of the study are the small size of the sample population and limited coverage of the brain with relatively thick slices which diminished our ability in detecting some small lacunar/subcortical lesions. The radiation dose is another concern. In our study, no patients showed acute radiation-related complications. The effective radiation dose in a typical CTP study is about 2 mSv (at 100–150 mA and 80 kV), which is significantly lower than xenon-perfusion CT and SPECT [15]. With advancement of new iterative reconstruction techniques [33], radiation dose of a CTP study can be reduced to fraction of the background radiation dose [34]. This would allow repeated examinations for suspected VCI patients with cerebral small vessel disease to monitor BBB permeability over time.

## Conclusion

This study demonstrated that with serial CTP imaging, a more profound decrease in CBF and CBV at acute phase and a higher BBB-PS in the non-infarcted basal ganglia and thalamus at subacute phase and month 3 in patients with lacunar/subcortical lesions, compared to patients with cortical stroke. These findings suggest that ischemic insult can exacerbate vascular abnormalities, especially subcortical BBB permeability in the presence of cerebral small vessel disease.

## Abbreviations

BBB: Blood–brain barrier
BBB-PS: Blood–brain barrier permeability surface product
CTP: CT perfusion
CBF: Cerebral blood flow
CBV: Cerebral blood volume
CSF: Cerebrospinal fluid
NECT: Non-enhanced CT
NIHSS: National Institutes of Health Stroke Scale
mRS: Modified Rankin scale
VCI: Vascular cognitive impairment
AD: Alzheimer’s disease
WML: White matter lesion
